# Efficient iterative virtual screening with Apache Spark and conformal prediction

**DOI:** 10.1186/s13321-018-0265-z

**Published:** 2018-03-01

**Authors:** Laeeq Ahmed, Valentin Georgiev, Marco Capuccini, Salman Toor, Wesley Schaal, Erwin Laure, Ola Spjuth

**Affiliations:** 1 Department of Computational Science and Technology, Royal Institute of Technology (KTH), Lindstedtsvägen 5, 10044 Stockholm, Sweden; 2 Department of Pharmaceutical Biosciences, Uppsala University, Box 591, 75124 Uppsala, Sweden; 3 Department of Information Technology, Uppsala University, Box 337, 75105 Uppsala, Sweden

**Keywords:** Virtual screening, Docking, Conformal prediction, Cloud computing, Apache Spark

## Abstract

**Background:**

Docking and scoring large libraries of ligands against target proteins forms the basis of structure-based virtual screening. The problem is trivially parallelizable, and calculations are generally carried out on computer clusters or on large workstations in a brute force manner, by docking and scoring all available ligands.

**Contribution:**

In this study we propose a strategy that is based on iteratively docking a set of ligands to form a training set, training a ligand-based model on this set, and predicting the remainder of the ligands to exclude those predicted as ‘low-scoring’ ligands. Then, another set of ligands are docked, the model is retrained and the process is repeated until a certain model efficiency level is reached. Thereafter, the remaining ligands are docked or excluded based on this model. We use SVM and conformal prediction to deliver valid prediction intervals for ranking the predicted ligands, and Apache Spark to parallelize both the docking and the modeling.

**Results:**

We show on 4 different targets that conformal prediction based virtual screening (CPVS) is able to reduce the number of docked molecules by 62.61% while retaining an accuracy for the top 30 hits of 94% on average and a speedup of 3.7. The implementation is available as open source via GitHub (https://github.com/laeeq80/spark-cpvs) and can be run on high-performance computers as well as on cloud resources.

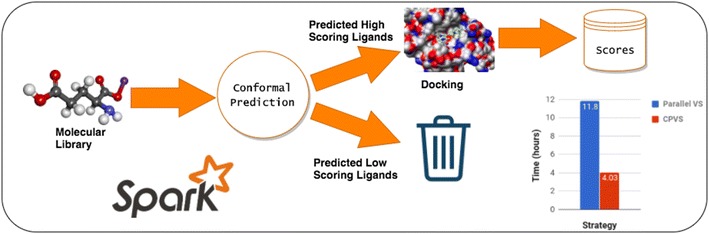

## Background

An important part of the drug discovery process is lead identification, where compounds that bind to a selected target protein are identified. A well-established approach for this is high-throughput screening (HTS), which includes screening a large number of chemical compounds against a target using an automated bioassay [1]. An alternative approach is in silico screening, where virtual chemical libraries are screened against a target receptor using computational methods [2–4]. A common method for this is molecular docking and scoring, where a docking algorithm is applied to find the best pose of the ligand in, e.g., the active site of a receptor, and a scoring function is used to evaluate the docking [5]. Virtual screening is trivially parallelizable on a per-ligand basis, and there have been many approaches developed for doing this [6].

Due to the recent availability of large molecule datasets (e.g., ZINC [7]) and their structure being highly parallelizable, parallel approaches have been used for virtual screening. In our previous studies [8, 9], we have shown that these large chemical libraries can be efficiently processed in parallel using Apache Spark [10] and scales well with increasing computation power. However, the docking step in virtual screening takes a notable amount of time even in a parallel setting. Also, only a small number of high-scoring ligands are found in these chemical libraries during the virtual screening process and much of time is wasted docking ‘low-scoring’ ligands. The docking time can be substantially reduced if high-scoring ligands can be inferred with confidence in advance so the ligands expected to be low-scoring can be skipped.

### Inference and machine learning

With the availability of large datasets in the last two decades, learning from data and extracting value from such large quantities of data has become a prominent field, generally known as machine learning. Supervised machine learning is the most common technique, where the aim is to derive a mapping from input *x* to output *y*, given a labeled set of input–output pairs [11]. The dataset is divided into training and test sets. Each input *x* in the training set is a vector of numbers representing some characteristics of the input, known as features. A model is trained using the training set and then used against the test set to get the predictions. The accuracy of the test set prediction is used to assess the model validity/performance.

Machine learning has been extensively utilized in a variety of fields and possesses nice theoretical properties. However, a common deficiency in conventional machine-learning algorithms is that they don't provide valid information about the reliability or confidence of the predictions made on the new examples [12]. The most common approach is to report and assume that a model will predict with comparable performance on future examples as it performed on the test examples. However, there is then an uncertainty that the new observation might be different from the test set, which has led to discussions and fuzzy definitions on a model's 'applicability domain'. What is desired is instead object-based confidence levels, and Conformal prediction is one such mathematical framework that gives valid confidence levels on predictions for each example, and answers the question: How good is your prediction?

### Conformal prediction

Conformal prediction is a method devised by Vovk et al. [13] that utilizes earlier knowledge to decide exact levels of confidence in new predictions. Conformal prediction can be used in combination with almost any underlying regression or classification algorithm, e.g., support-vector machines, gradient boosting, neural networks, and random forests. In the case of classification models, conformal prediction produces a set of labels, e.g., in binary classification it produces {0}, {1}, {0, 1} and { } sets. Although the output is a region or multi-classed rather than a point prediction, the main benefit of the technique is the model validity with user-provided confidence threshold. For example, in a binary classifier the true label is on average not excluded more than the confidence threshold, e.g., if the confidence level is 90%, then in 10% of the cases the true label will be excluded.

One of the basic setups for conformal prediction is the transductive approach. In this scenario, the model is retrained for each new observation. However, this is quite computationally expensive especially for problems with large datasets and therefore an inductive or batch setting has become popular, called Inductive Conformal Prediction (ICP) [14].

The way ICP works in a classification setting is fairly simple. Initially, a training set and a test set of examples with labels is required. The training set is divided into a proper training set and a calibration set. The training set is used to train a model using any underlying algorithm. The calibration set is used to measure a *nonconformity score* for each observation in the calibration set, which is a measure of how different the current example is compared to the training set. The model is then used to predict the examples in the test set, and for each class label $l = 1, \ldots , k$, a p-value of *x* for class *l* is computed. If the p-value for class label *l* is greater than ${\upvarepsilon }$, it is added to the prediction set. Using this strategy, it is guaranteed that on average the true label of *x* will be present in the prediction set with probability $1-{\upvarepsilon }$ [14].

Conformal prediction has been successfully used for moderate to small datasets in quantitative structure-activity relationship (QSAR) predictive modeling [15, 16], complication risk prediction following a coronary drug eluting stent procedure ($\sim$ 2 K examples) [17], and anomaly detection of trajectories [18]. In a recent study by Svensson et al. [19], a conformal prediction based iterative approach is proposed for efficient screening. Docking was performed on an initial small dataset and then conformal predictors were used to find active molecules in an iterative fashion.

### Apache Spark and MLlib

Apache Spark [10] is a parallel programming and execution framework for cluster computing that is fast and easy to use. In terms of speed, it's much faster than the well-known Google MapReduce [20] and its open source implementation, Apache Hadoop. One reason for its agility is keeping the data in-memory with support for iterative processing. A detailed discussion is provided in our earlier work [8, 9] on choosing Spark for parallel virtual screening in comparison to other parallel frameworks, such as OpenMPI, MPI and Google MapReduce.

Another advantage of Spark is the scalable machine learning library, MLlib. MLlib includes many machine learning algorithms such as classification, regression, clustering and collaborative filtering and useful tools such as featurization, machine learning pipelines, statistics and linear algebra utilities. It is an open source project and has a rapidly growing community of developers. It has been successfully used for various parallel machine learning projects, e.g., Capuccini et al. [21] presents an MLlib-based distributed conformal prediction implementation for valid confidence estimation for large dataset problems and shows the validity and scalability of the algorithms using two large datasets.

Here we present a novel strategy for distributed structure-based virtual screening using Spark's MLlib library, distributed conformal prediction [21] and support vector machines (SVM) [22]. The objective is to avoid docking molecules that can be predicted as ‘low-scoring’ ligands with a certain confidence. To achieve this we dock a subset of molecules iteratively and the conformal predictor is re-trained until the model reaches a certain efficiency level, whereafter all remaining ligands predicted as 'high-scoring' are docked. Our results show that with this strategy we are able to dock much fewer molecules than in normal virtual screening while retaining a high sensitivity.

## Methods

### Data

We used the SureChEMBL molecule library [23] for our benchmarks, downloaded from ZINC [7] in ready-to-dock SDF format. The library contains $\sim$ 2.2 M molecules and takes $\sim$ 8 GB of disk space. Molecules were described using the signature molecular descriptor [24], which is a 2D graph based on the signature of atoms in the molecule, where an atom signature is a representation of the atom's local environment in terms of neighboring atoms up to a specified distance (height). We used a parallel spark based implementation of the signature descriptor [25] and set the consecutive signature heights 1–3, i.e., an atom at a distance of max 3 edges. An earlier study [26] suggests that signature height 1–3 produces good results for molecular classification with SVM based models. OEDocking TK [27] was used as the underlying docking software and as target proteins for the docking we chose the HIV-1 protease [28], PTPN22, MMP13 and CTDSP1 [29].

### Analysis workflow

**Fig. 1:**
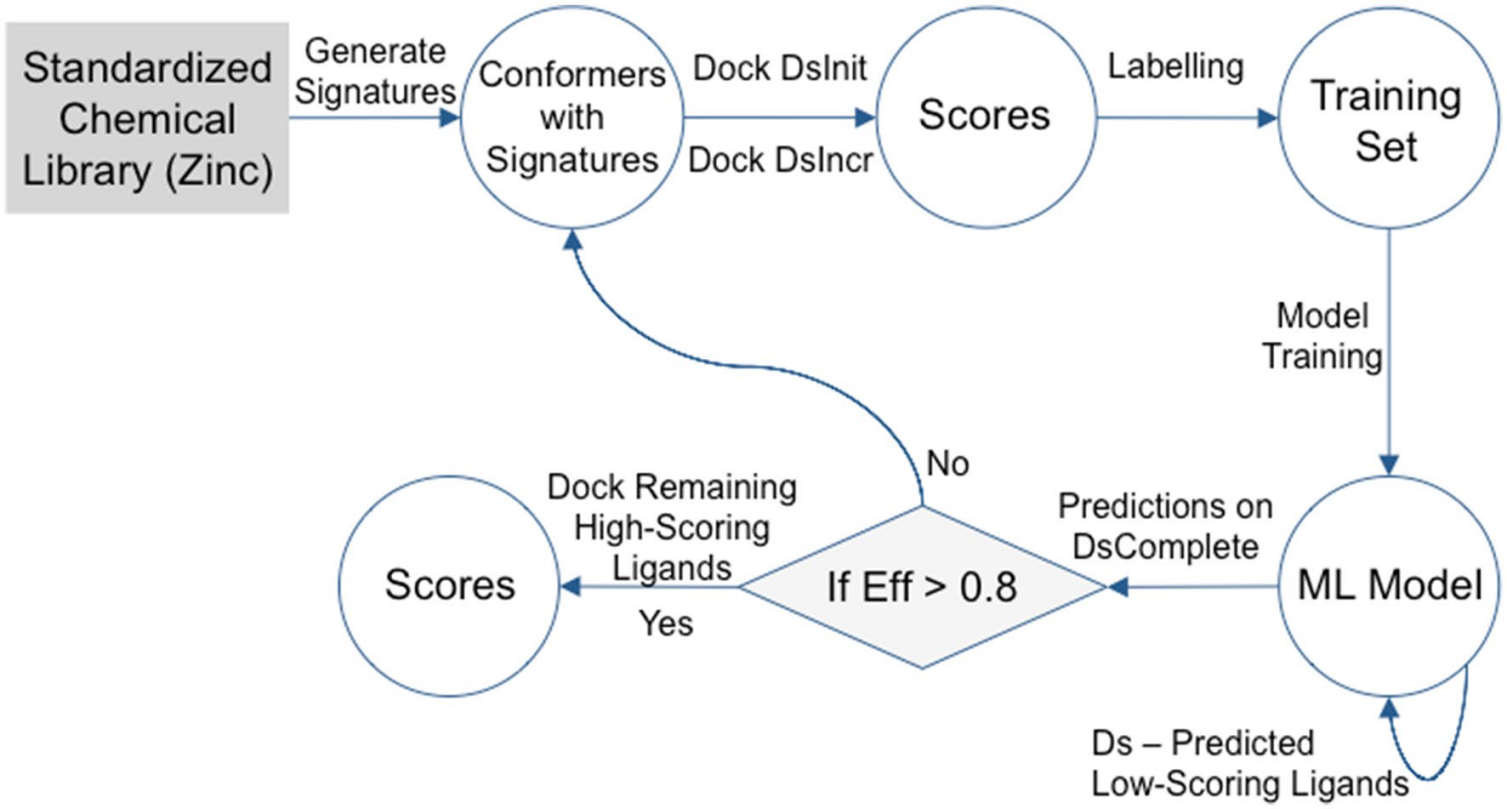
Workflow of CPVS. Signatures were generated for the whole dataset with two copies named *Ds* and *DsComplete*. An initial sample of *DsInit* number of molecules was randomly taken from *Ds* and docked against a chosen receptor and scores were calculated. To form a training set, docking scores were converted to class labels {0} and {1} representing ‘low-scoring’ and ‘high-scoring’ ligands, respectively. This was done using a 10-bin histogram of the docking scores where labels were assigned to ligands in different bins. An SVM-based conformal predictor model was trained on the training set and predictions were made on the whole Dataset *DsComplete*. The molecules were classified as ‘low-scoring’ ligands {0}, ‘high-scoring’ ligands {1} and 'unknown'. The predicted ‘low-scoring’ ligands were removed from *Ds* in each iteration and were hence never docked. Model efficiency was computed by finding the ratio of single label predictions [30], i.e., {0} and {1} against all predictions. The process was then repeated iteratively with a smaller data sample *DsIncr* from *Ds* which was docked and labeled, and the model was re-trained until it reached an acceptable efficiency. Thereafter all remaining ‘high-scoring’ ligands were docked. The scores of all docked molecules were sorted and accuracy for top 30 molecules was computed against the results from an experiment where all molecules were docked [9]

The objective of conformal prediction based virtual screening (CPVS) is to reduce total time by avoiding the docking of molecules that can be predicted as ‘low-scoring’ ligands and only dock compounds that are predicted as ‘high-scoring’ ligands with a certain confidence. The workflow is shown in Fig. 1.

Initially, signatures were calculated for all molecules in the whole dataset, and two copies of it were made: *Ds* and *DsComplete*. An initial sample of *DsInit* number of molecules was randomly taken from *Ds* and docked against a chosen receptor and scores were calculated. To form a training set, docking scores were converted to class labels {0} and {1} representing ‘low-scoring’ and ‘high-scoring’ ligands, respectively. This was done using a 10-bin histogram of the docking scores where labels were assigned to molecules in different bins. A conformal predictor was trained on the training set and predictions were made on the whole dataset, *DsComplete*. The molecules were classified as ‘low-scoring’ ligands {0}, ‘high-scoring’ ligands {1} and ‘unknown’, i.e., both lables {0, 1} or empty {}. The predicted ‘low-scoring’ ligands were removed from *Ds* in each iteration and were hence never docked. Model efficiency was computed by finding the ratio of single label predictions [30], i.e., {0} and {1} against all predictions. The process was then repeated iteratively with a smaller data sample *DsIncr* from *Ds*. The predictor was re-trained until it reached an acceptable efficiency, and all remaining ‘high-scoring’ ligands were docked. The scores of all docked molecules were sorted and accuracy for top 30 molecules was computed against the results from an experiment where all molecules were docked [9].

#### Modeling

We used a mondrian inductive conformal prediction (ICP) approach with SVM as underlying modeling method, a widely-used machine learning algorithm for predictive modeling [31, 32]. We used linear SVM, which has previously shown good results for QSAR modeling [33, 34], and used the implementation in Spark MLlib with L-BFGS for optimization because it works well with imbalanced datasets. A maximum of 50 iterations were used for L-BFGS optimization. The training set was randomly divided into 10% as calibration set and 90% as proper training set and the confidence level was set at 80%, which has been shown to work well in earlier studies with imbalanced datasets [35].

## Results

In order to tune our workflow, a number of parameters need to be selected in order to reduce the overall time for the virtual screening. This includes minimizing the number of docked molecules and keeping the size of training sets used for modeling as small as possible to avoid overly time-consuming training.

### Initial training set and labeling strategy

A critical component in the analysis is the first predictive model, and the initial training set must be of sufficient size to produce robust results with a minimum of false positives. The sizes of initial training set *DsInit* tested were 50, 100, 200 and 300 K.

**Fig. 2:**
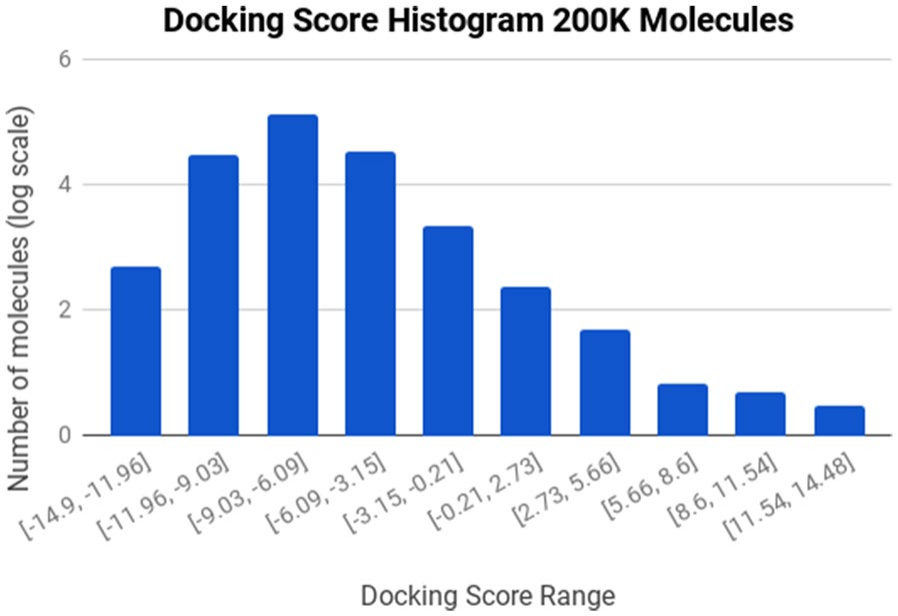
Docking score histogram for 200 K ligands shows an example docking score histogram for a sample of 200 K ligands in log scale. The data distribution is skewed right because we have fewer molecules with high scores, which is normal for these types of datasets as only a few ligands have a good fit with the target protein and the majority will not bind with high affinity

Docking scores were divided into 10-bin histograms, where some bins were assigned as ‘low-scoring’ or ‘high-scoring’. Four combinations were evaluated: 1_6, 1_5, 1_4, 2_4, where the first number is the highest bin for ‘low-scoring’ and the second number is lowest bin for ‘high-scoring’ ligands. For example, 2_4 declares that bins 1 and 2 contain ‘low-scoring’ ligands while bins 4 through 10 contain ‘high-scoring’ ligands. The unassigned bin 3 is excluded from training. Figure 2 shows an example docking score histogram for a sample of 200 K ligands in log scale. The data distribution is skewed because we have fewer molecules with high scores, which is normal for these types of datasets as only a few ligands have a good fit with the target protein and the majority will not bind with high affinity.

The labeling of the initial sample of *DsInit* as ‘low-scoring’ ligands needs to contain as few (observed) high-scoring binders as possible, hence the number of bins selected as class 0 should be kept low. The labeling of ‘high-scoring’ ligands should minimize the chance of not including (observed) high-scoring binders, hence the number of bins selected as class 1 should be kept high. This formed the basis for choosing the evaluated bin combinations (see Table 1).

**Table 1:** Effect of *DsInit* size and bin combination on accuracy and efficiency for the initial trained model (repeated 10 times)

Trail no.	*DsInit* (K)	Bins	Accu. (avg)	Accu. (SD)	Eff. (avg)	Eff. (SD)
1	50	1_6	45.33	47.22	65	23
2	50	1_5	65.33	43.95	63	23
3	50	1_4	78.34	41.31	44	17
4	50	2_4	94.34	4.46	79	18
5	100	1_6	89.67	6.37	73	16
6	100	1_5	94.67	5.92	75	18
7	100	1_4	88.34	29.91	31	12
8	100	2_4	89.67	7.45	91	11
9	200	1_6	93.00	3.99	65	15
10	200	1_5	96.34	1.89	76	17
11	200	1_4	97.67	2.25	43	20
12	200	2_4	90.34	9.74	91	6
13	300	1_6	86.67	8.01	44	12
14	300	1_5	95.34	4.50	63	17
15	300	1_4	98.34	1.76	54	22
16	300	2_4	86.00	7.17	94	5

Table 1 shows the effect of the different combinations of *DsInit* size and labeling parameters on accuracy and efficiency after the first iteration. Each run was repeated 10 times and the average and standard deviation for accuracy and efficiency was computed. In general, increased efficiency and accuracy was reported with increased size of *DsInit*, but the labeling strategy based on bins combination also affected the results. Runs with *DsInit* size 50 and 100 K were discarded because of the risk of fluctuation in the first model due to sampling issues with smaller datasets, observable by higher variance in the accuracy. For the remaining runs, the best combination of high model accuracy and efficiency was sought. Higher accuracy of the initial model reduces the chances of discarding actual high-scoring binders, and higher efficiency implies fewer iterations to reach sufficient model efficiency in the iterative model building. We selected run 10 in Table 1, i.e., the parameters with *DsInit* size 200 K and bins 1_5, which had a mean accuracy of 96.34% and an efficiency of 76%.

### Incremental model building

Improving the efficiency of the model in each iteration requires sufficient amount of new data added to the training set. Table 2 shows the effect of *DsIncr* size on accuracy and model efficiency. We evaluated values 50, 100 and 200 K for *DsIncr* and ran the iterative implementation until the desired efficiency was reached. Each run was performed 20 times. Accuracy and efficiency of the final models in all three setting were good and similar to each other. In terms of time consumption, a *DsIncr* size of 100 K required the least total time to complete. The two core factors that contribute to the total time are the number of docked molecules and the time used for model training and predictions. The number of molecules docked for all three settings were rather similar, i.e, $\sim$ 0.8 million. In all three settings, the model eventually reached the required 80% efficiency though the smaller *DsIncr* needed more iterations. With *DsIncr* size as 50 K, an average of 3.90 models needed to be trained whereas with *DsIncr* size as 200 K, although we need to train only 3.15 models, each model training takes more time because of larger size of data. Based on this argumentation, *DsIncr* size was set to 100 K for the final runs.

**Table 2:** Selecting *DsIncr* size for incremental model building (repeated 20 times, mean values reported)

*DsIncr* (K)	Iterations	Accu.	Eff.	Docked mols (millions)	Total time (relative)
50	3.9	96.5	0.91	0.77	1
100	3.35	96.84	0.91	0.81	0.96
200	3.15	97.17	0.91	0.79	1.12

Paremeters *DsInit* size = 200 K and Bins = 1_5 for all runs. Time was calculated relative to 50 K

### Efficiency of CPVS

We evaluated the performance of CPVS in terms of reduction of total time, benchmarked against our previous study [9] (referred to as PVS) where the same dataset was processed in the same parallel fashion but without the machine learning component to filter out ‘low-scoring’ leads.

#### Experimental environment

A standalone Spark cluster, along with HDFS was launched on the SNIC Science Cloud (SSC) [36] using SparkNow [37] for automated image creation and initiating required services on virtual machines. A total of 12 nodes were launched each with 8 virtual CPUs (vCPUs), 16 GB of RAM, 160 GB of disk storage and 40 GB of block storage. It was a completely virtualized environment and in that sense similar to commodity computing based clusters. One node was used as the Spark driver, which did not take part in processing. The remaining 11 nodes were used as workers with a total of 88 cores.

#### Benchmarking

**Fig. 3:**
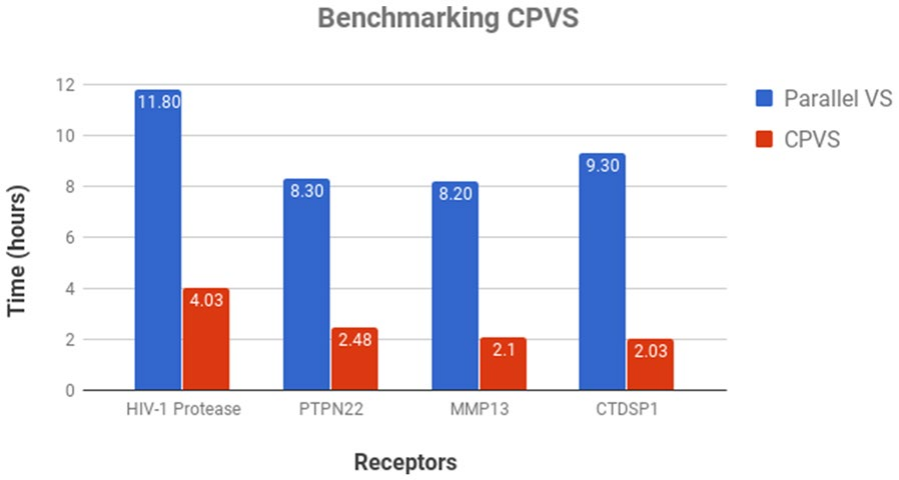
Benchmarking CPVS against parallel VS. On average, only 37.39% of the ligands were docked to reach an accuracy level of $\sim$ 94%. By decreasing the number of docked molecules, CPVS saves more than two-thirds of the time and got an average speedup of 3.7 in comparison to Parallel VS [9]

As summarized in Fig. 3, both the PVS and CPVS runs were executed on the same computational infrastructure and the time for job completion was recorded. PVS, performing an exhaustive search, was executed once and took 11.8, 8.30, 8.20 and 9.30 hours to complete against HIV-1, PTPN22, MMP13 and CTDSP1 receptors respectively.

CPVS was executed 10 times for each target receptor and the results are given in Table 3. For all four receptors, CPVS completed at least three times faster than PVS and the accuracy was at least 90%. The average accuracy for all four receptors is $\sim$ 94%. In general, the variance in results was low showing that the results were consistent. The average speedup (PVS total time / CPVS total time) for four receptors was computed to 3.7.

**Table 3:** Results of the CPVS method for a set of target receptors

Receptor	Iterations	Accu.	Docked mols (%)	Time (hours)	Speed up
HIV-1	3.9	97.33	37.15	4.03	2.93
PTPN22	4.7	98.34	44.77	2.48	3.35
MMP13	3.5	89.00	33.34	2.10	3.90
CTDSP1	3.6	92.67	34.29	2.03	4.58

Results were averaged over 10 runs for each receptor

## Discussion

The docking step makes structure-based virtual screening a compute intensive task that requires high-performance clusters or cloud computing resources to complete in a timely manner. Our iterative virtual screening methodology using conformal prediction to filter out molecules from the actual docking shows effective results in that on average only 37.39% of the ligands were docked to reach an accuracy level of $\sim$ 94% based on the top 30 top binders, and saving about two-thirds of the total computation time. These results complement the earlier study by Svensson et al. [19] who showed that 57% of the active compounds could be found by only docking 9.4% of the compounds using the DUD ligand set of 2950 compounds using a conformal prediction approach. In CPVS we use a more realistic screening dataset of over 2.2 M compounds, and the stepwise iterative docking and machine learning on such a large dataset was facilitated by the use of Apache Spark for distributed computations and would have been complex and inefficient to carry out without a distributed data framework.

Some common data manipulation operations can be quite expensive even in a distributed environment as it could lead to a lot of data shuffling among the nodes. For labeling purposes, the histogram approach was used to tackle one such problem. Another straightforward approach could have been to compute the top and bottom percentiles but this would include initial sorting of the data based on scores which is an expensive operation in a distributed environment. Thus a lighter histogram operation was utilized, which also showed good results.

While the major advantage of the method is to shorten the virtual screening execution time, it also opens up opportunities for large-scale studies which may involve multiple target receptors and multiple large molecule libraries. The ability to execute the analyses in parallel on HPC and cloud resources makes it only limited by resources and/or costs. The instantiation of Apache Spark clusters on-demand has been a complex task earlier, but it is nowadays a straightforward operation on the major cloud providers, and there are frameworks developed that greatly simplifies this process on private clouds (e.g., SparkNow [37]) or in HPC environments (e.g., spark-on-slurm [38], sparkhpc [39]).

Processing large datasets is time consuming and costly in that it requires large compute infrastructures to complete jobs within reasonable time. This limited our opportunity for parameter sweeps in the study and necessitated a more tailored approach. We also note that our results depend on the docking time and hence the docking implementation (OEDocking TK in our case). However we do not believe that major changes to parameters will be required in order to reach an efficient iterative docking with machine learning for other docking toolkits.

## Conclusion

In this work we present an efficient methodology for parallel virtual screening using conformal prediction to filter out ‘low-scoring’ ligands and only dock molecules that are predicted as 'high-scoring' ligands with a specified accuracy. We were able to reduce the number of docked molecules by 62.61% while retaining $\sim$ 94% accuracy for the top 30 binders. The CPVS average total time for each receptor was at least 3 times less than for PVS [9] on the same dataset in the same computational environment. This makes CPVS a vital and cost effective alternative for parallel virtual screening. The source code of the implementation is publicly available on GitHub (https://github.com/laeeq80/spark-cpvs).
